# Acute cerebrovascular disease in the Philippine Neurological Association One Database (PNA1DB)—patient profiles and disparities between public and private hospitals

**DOI:** 10.3389/fstro.2026.1835562

**Published:** 2026-06-29

**Authors:** Robert N. Gan, Jose Leonard R. Pascual V, Maria Epifania V. Collantes, John Harold B. Hiyadan, Dan Neftalie A. Juangco, Ma. Cristina Z. Macrohon-Valdez, Cyrus G. Escabillas, Christian Oliver C. Co, Gemmalynn B. Sarapuddin, Maria Teresa A. Cañete, Raquel M. Alvarez, Belinda L. Mesina-Nepomuceno, Johnny K. Lokin, Marie Charmaine Sy Lukban, Rosalina E. Picar

**Affiliations:** 1 Disease Study Management Group, Philippine Neurological Association One Database-Stroke, Quezon City, Philippines; 2 Philippine General Hospital, University of the Philippines Manila, Manila, Philippines; 3 Baguio General Hospital and Medical Center, Baguio, Philippines; 4 East Avenue Medical Center, Quezon City, Philippines; 5 St. Luke's Medical Center, Quezon City/Global City, Philippines; 6 Jose R. Reyes Memorial Medical Center, Manila, Philippines; 7 Quirino Memorial Medical Center, Quezon City, Philippines; 8 The Medical City, Pasig, Philippines; 9 Chong Hua Hospital, Cebu City, Philippines; 10 Makati Medical Center, Makati City, Philippines; 11 University of the East—Ramon Magsaysay Memorial Medical Center, Quezon City, Philippines; 12 University of Santo Tomas Hospital, Manila, Philippines

**Keywords:** disparities, lower-middle income country, Philippines, quality of healthcare, registry, stroke

## Abstract

**Background:**

Stroke is a leading cause of death in the Philippines, yet data on epidemiology, care, and outcomes remain scarce. We aimed to describe the caseload, patient profile, management, and outcomes of acute cerebrovascular events, and to assess disparities between publicly funded and privately funded tertiary hospitals in the Philippines.

**Methods:**

The PNA1DB-Stroke project is a prospective, multicenter, registry of consecutive patients aged ≥18 years admitted with transient ischemic attack (TIA), ischemic stroke, hemorrhagic stroke, or cerebral venous thrombosis (CVT) in 11 (five publicly-funded, six privately-funded) accredited neurology training tertiary hospitals across the Philippines. Data on socio-demographics, medical history, event type, clinical assessments, diagnostic procedures, treatments and discharge outcomes were collected from June 1, 2021, to August 31, 2024.

**Results:**

Among the 15,230 cases included, mean age 58.0 ± 14.2 years, 6,726 (44.2%) women, hemorrhagic strokes accounted for 38.6%. A total of 10,974 (72.1%) cases were admitted in public hospitals with a larger proportion of hemorrhagic strokes compared to private hospitals (*p* < 0.001). Public hospitals cases were younger, socioeconomically disadvantaged, and more often have lifestyle risk factors. Only 620 (9.3%) ischemic stroke cases underwent revascularization. While relatively more cases received thrombolysis in public hospitals, major diagnostic and therapeutic procedures were performed less compared to private hospitals. Overall, in-hospital fatality was 16.4%, higher in public hospitals (20.5%) with worse neurological outcomes at discharge than in private hospitals.

**Conclusions:**

Our registry demonstrated a high burden of hemorrhagic stroke, delayed hospital arrival, underutilization of reperfusion therapies, and striking disparities between public and private hospitals in the PNA1DB. Study Registration: ClinicalTrials.gov NCT04972058.

## 1 Introduction

Stroke is the third leading cause of mortality in the Philippines, accounting for approximately 60,000 deaths annually or 10% of all deaths nationwide (Philippine Statistics Authority, 2025). Despite this substantial burden, the true national prevalence of stroke is uncertain, with published estimates varying widely from 0.486 to 6% depending on study design and population sampled (Collantes et al., 2022a; Navarro et al., 2014). This variability underscores persistent gaps in stroke surveillance and epidemiologic reporting in the country. Nonetheless, the high mortality rate associated with stroke reflects the widespread prevalence of risk factors such as hypertension, diabetes, smoking, and obesity (Sy et al., 2012). These clinical risks are compounded by systemic barriers such as poor public health awareness of stroke symptoms, delays in seeking medical attention, limited number of stroke ready hospitals, inadequate facilities and rehabilitation resources, especially in rural and underserved regions. Collectively, these factors contribute to delayed presentation, suboptimal acute management, and reduced opportunities for recovery (Collantes et al., 2021, 2022a).

Socioeconomic factors, such as financial resources, geographical locations and limited government support and programs continue to be major challenges in medical care (Collantes et al., 2021). The provision of healthcare in the country is largely dichotomized between privately funded facilities (“Private”), which are managed by corporations with investors and preferred by patients with financial capability to fully bear the cost out-of-pocket for quicker services, better facilities, and often in a more comfortable environment, and publicly funded facilities (“Public”), which are supported and subsidized yearly by the limited budget allocated by the government with patients and relatives bearing the excess cost. The devolution of the Philippine health system has further contributed to health care fragmentation, lack of technical coordination and misappropriation of health budget by some local government units (Cuenca, 2018).

Although national policies and early stroke awareness efforts have been implemented, profound challenges persist, including data insufficiency, inequitable access, and high treatment costs. The scarcity of systematic collection of reliable data precludes any determination of the basic profile, outcomes and quality of care received by stroke patients. This lack of comprehensive, standardized data limits meaningful benchmarking across institutions and hinders evaluation of existing stroke care initiatives. Reliance on very few published local studies fail to reflect the full spectrum of the real-world needs, thus providing little guidance for the development of models of care based on identified potential areas for improvement.

To address these gaps, the Philippine Neurological Association One Database-Stroke (PNA1DB-Stroke) project was initiated by the Philippine Neurological Association (PNA) as a platform to systematically collect data on acute cerebrovascular diseases that will support data-driven recommendations to guide public health policies, direct resources to areas in need and ultimately improve overall patient outcomes. In this study, we describe the caseload, profile, types of diagnostic and therapeutic management, and outcomes at discharge of cases presenting with acute cerebrovascular events, i.e., transient ischemic attack (TIA), ischemic and hemorrhagic stroke, and cerebral venous thrombosis (CVT) in 11 representative tertiary hospitals in the Philippines. We additionally examined disparities in management and outcomes of cases between public and private hospitals.

## 2 Methods

### 2.1 Design

PNA1DB-Stroke is a pragmatic, multi-center, prospective, observational study and standing database of patients hospitalized for TIA or stroke (ClinicalTrials.gov NCT04972058). The protocol was previously published and is described here briefly (Philippine Neurological Association One Database-Stroke, 2022).

### 2.2 Study population and setting

In 2021, there were 11 accredited adult neurology residency training hospitals in the Philippines. The PNA1DB project was implemented in these tertiary hospitals because of feasibility and availability of operational support. The participating hospitals are a mixture of publicly funded (Baguio General Hospital, East Avenue Medical Center, Jose R. Reyes Memorial Medical Center, Quirino Memorial Medical Center, Philippine General Hospital) and privately funded hospitals (Chong Hua Hospital, Makati Medical Center, St. Luke's Medical Center, The Medical City, University of Santo Tomas Hospital, University of the East—Ramon Magsaysay Memorial Medical Center). While not necessarily representative of all hospitals in the archipelagic country, the hospital type (public or private) may act as proxy for multiple patient-level, institutional, and healthcare factors, reflecting the care received by majority of stroke patients.

All patients aged ≥18 years old with first or recurrent TIA, ischemic or hemorrhagic stroke, or CVT who were admitted in any of the 11 tertiary hospitals between 1 June 2021 to 31 August 2024 were included. A clinical diagnosis of TIA or stroke by a neurologist was required for inclusion in the database based on the general standard definition of TIA and stroke. Classifying the case as TIA, ischemic or hemorrhagic stroke or CVT was based on brain imaging. We excluded cases who were admitted to the hospital for medical conditions other than an acute cerebrovascular event, e.g., admitted for pneumonia, rehabilitation, or procedures, such as gastric tube insertion, etc.

### 2.3 Data collection

Each hospital maintained a daily census that included personal information of every new patient admitted at their facility. Based on the logs, each case was assigned a case identification number. Anonymized, uniform, pre-defined set of data fields on socio-demographics, medical history, index event type, clinical assessments, in-hospital diagnostic and therapeutic management, and discharge outcomes were collected and encoded into a secure online case report form (eCRF) prospectively for each case as they were admitted and discharged. Outcomes assessed at discharge included length of hospital stay (LOS), National Institute of Health Stroke Scale (NIHSS), Glasgow Coma scale (GCS) and modified Rankin Scale (mRS). No post-discharge visit was required.

All site study team members were trained on the protocol and the eCRF during investigators meetings at the start and midway through the study. Trainings were recorded and made available for refresher training by study personnel. Access to database entry was provided to new study team members only after confirmation by the principal investigator that training was completed.

As ethic committee/institutional review board (EC/IRB) approval of each hospital was not all received at the same time, actual start dates of data collection at each hospital were staggered. Nevertheless, each hospital provided data over a 3-year period. To reduce data entry errors, system checks were built into the eCRF, while data integrity and completion checks were run regularly. Full database lock was achieved on July 21, 2025.

### 2.4 Ethical considerations

The study was guided by the principles of Good Clinical Practice, the National Ethical Guidelines for Health and Health-Related Research 2017, and in accordance with local regulations (U.S. Department of Health and Human Services, Food and Drug Administration, Center for Drug Evaluation and Research (CDER), Center for Biologics Evaluation and Research (CBER), 2021, Philippine Health Research Ethics Board, 2018). The protocol was approved by the Single Joint Research Ethics Board (SJREB) of the Department of Health (SJREB-2021-20), as well as by the EC/IRB of each hospital.

As an observational study that intended to capture all cases over the study period and (1) involves no more than minimal risk to subjects, (2) the principal risk of harm to the subject would be a breach of confidentiality, and (3) the subject's signature on the informed consent document will be the only record linking the subject to the research, a waiver of informed consent was approved by the SJREB. A standardized information sheet was provided to all patients, but written informed consents were obtained at two hospitals as required by their local EC/IRB.

### 2.5 Statistical analyses

Baseline socio-demographics and medical history were summarized for the overall population and further stratified to examine disparities between public and private hospitals. Continuous variables were expressed as mean ± standard deviation (SD) or median with interquartile range (IQR) as appropriate, while categorical variables (Supplementary Table 1) were presented as absolute counts and percentages. Missing data were managed using pairwise deletion without statistical imputation. Univariable analysis between public and private hospitals were evaluated using Pearson's Chi-square tests for proportions and independent *t*-tests for continuous measures. A two-tailed *p*-value < 0.05 was considered statistically significant.

To examine disparities in the utilization of diagnostic and therapeutic procedures between public and private hospital clusters, we utilized log-linear Modified Poisson regression models with robust standard errors. This approach was selected to provide Adjusted Relative Risks (aRR) and corresponding 95% confidence intervals (CI), as odds ratios may overestimate effect sizes in large cohorts where the outcomes are common. Private hospitals served as the reference group for all comparative analyses.

Three sequential multivariable models were constructed to assess potential confounding: Model 1 adjusted for age and sex; Model 2 further adjusted for stroke severity on admission (NIHSS and GCS); and Model 3 represented the fully adjusted model, incorporating covariates from Model 2 plus annual household income and onset-to-hospital arrival time.

Outcomes were adjusted for age, sex, admission NIHSS and GCS, transfer status, onset-to-arrival time, and income. Linear Mixed-Effects Model was used for continuous outcome measures, treating hospital type as a fixed effect and individual hospitals incorporated as a random intercept to control for hospital-level clustering, and Mixed-Effects Logistic Regression with hospital as random intercepts for categorical measures.

All statistical analyses were performed using R version 4.3.1 (R Foundation for Statistical Computing, Vienna, Austria). Multivariable frameworks were computed via the base glm() function, while robust variance-covariance parameters were extracted using the sandwich and lmtest packages. Data management and table formatting were facilitated using the dplyr, readr, officer, and flextable packages (R Core Team, 2025; Wickham et al., 2024; Warnes et al., 2024; Venables and Ripley, 2002).

## 3 Results

### 3.1 Caseload

**Figure 1:**
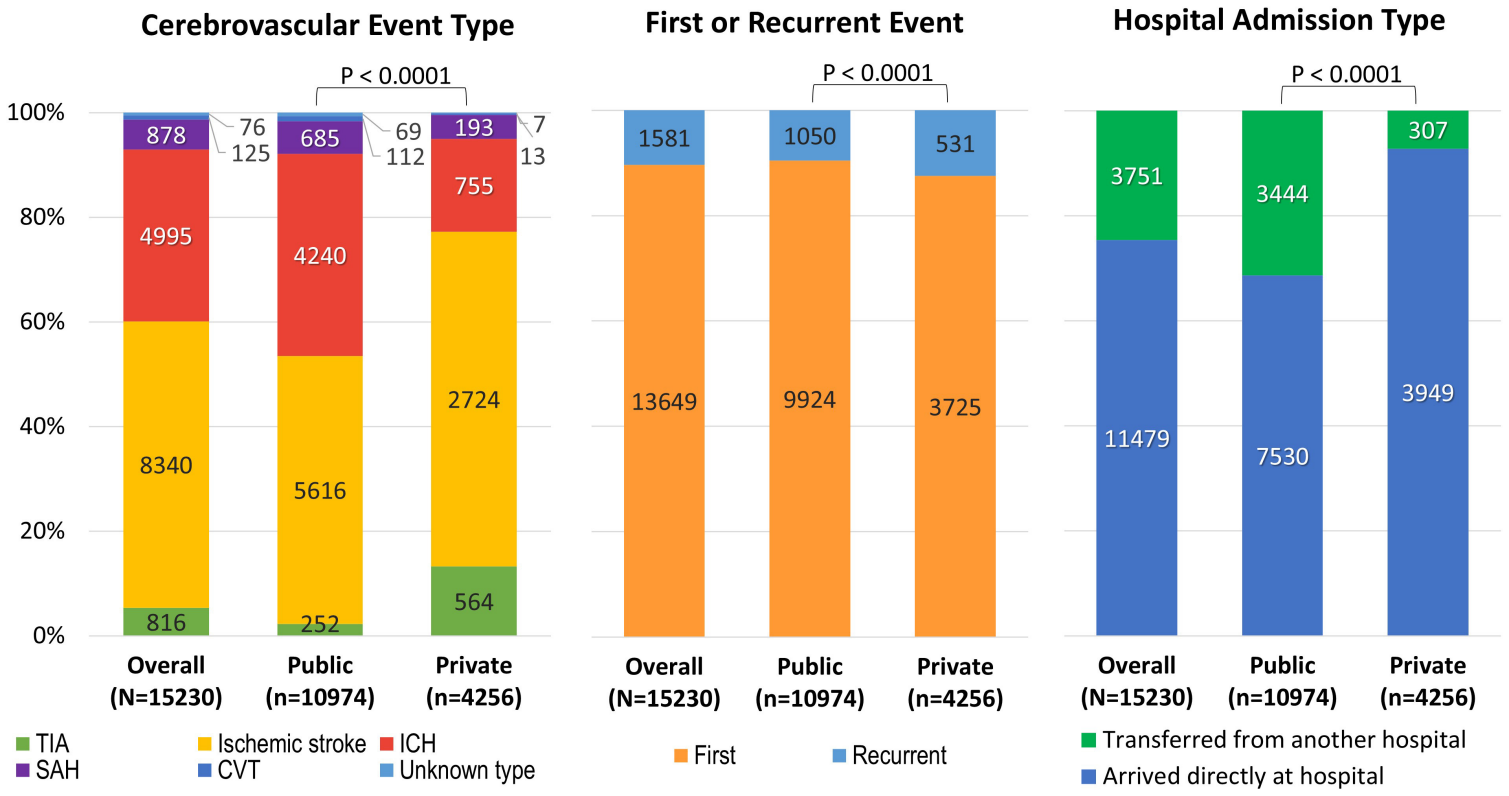
Caseload of acute cerebrovascular events in the PNA1DB-Stroke overall and by types of hospital, event, and admission. CVT, cerebral venous thrombosis; ICH, intracerebral hemorrhage; SAH, subarachnoid hemorrhage; TIA, transient ischemic attack.

All 15,230 hospitalized cases of clinically diagnosed TIA, stroke and CVT were included in the database over a 3-year period, of which 10,974 (72.1%) were admitted in public hospitals and 4,256 (27.9%) in private hospitals (Figure 1). Overall, 9,156 (60.1%) of cases were TIA or ischemic stroke while 5,873 (38.6%) were hemorrhagic strokes, i.e., intracerebral hemorrhage (ICH) or subarachnoid hemorrhage (SAH). CVT accounted for only 125 (0.8%) while only 76 (0.5%) were of unknown stroke type due to patients dying, absconding or being discharged before any brain imaging was performed. The proportion of hemorrhagic strokes was remarkably higher in public than private hospitals (*p* < 0.0001). A history of previous stroke was reported in 1,581 (10.4%) cases, which was more frequent in private hospitals. Participating hospitals received 3,751 (24.6%) cases of transfers from another hospital, largely by public hospitals (*p* < 0.0001).

### 3.2 Profile of cases

The overall study population had a mean age of 58.0 ± 14.2 years and included 6,726 (44.2%) women (Table 1). Hypertension (65.6%) was the most common risk factor. Most common mechanistic cause was intracranial large-artery atherosclerosis (33.2%) for ischemic stroke, hypertensive (88.9%) for ICH, and aneurysmal (94%) for SAH. Stroke severity on admission, as measured by NIHSS and GCS, was worse among hemorrhagic strokes compared to ischemic stroke.

**Table 1:** Profile of acute cerebrovascular cases in the PNA1DB-stroke overall and by type of hospital.

Characteristics	Overall (*N* = 15,230)	Public (*n* = 10,974)	Private (*n* = 4,256)	*p*-value
Age, years (mean ±*SD*)	58.0 ± 14.2	56.8 ± 13.6	61.1 ± 15.3	< 0.0001
Sex (***n*****, %)**				< 0.0001
Male	8,503 (55.8)	6,315 (57.5)	2,188 (51.4)	
Female	6,726 (44.2)	4,658 (42.4)	2,068 (48.6)	
Trans-male	1 (0)	1 (0)	0 (0)	
Ethnicity–Filipino (*n*, %)	15,117 (99.3)	10,966 (99.9)	4,151 (97.5)	< 0.0001
Occupation (*n*, %)	(*n* = 12,678)	(*n* = 8,851)	(*n* = 3,827)	< 0.0001
Employed	5,088 (40.1)	3,527 (39.8)	1,561 (40.8)	
Homemaker	3,309 (26.1)	2,621 (29.6)	688 (18)	
Retired	1,972 (15.6)	906 (10.2)	1,066 (27.9)	
Currently unemployed	1,990 (15.7)	1,548 (17.5)	442 (11.5)	
Never worked	319 (2.5)	249 (2.8)	70 (1.8)	
Migrant from another region (*n*, %)	4,364 (28.7)	3,777 (34.4)	587 (13.8)	< 0.0001
Approximate annual household income (*n*, %)	(*n* = 14,577)	(*n* = 10,963)	(*n* = 3,614)	< 0.0001
Under Php 250k (approx. $4,400)	9,379 (64.3)	8,553 (78.0)	826 (22.9)	
**Known past history of vascular risk factors (** ***n*** **, %)**				
Hypertension	9,991 (65.6)	6,852 (62.4)	3,139 (73.8)	< 0.0001
Diabetes mellitus	3,015 (19.8)	1,524 (13.9)	1,491 (35)	< 0.0001
Dyslipidemia	1,700 (11.2)	738 (6.7)	962 (22.6)	< 0.0001
Atrial fibrillation	552 (3.6)	245 (2.2)	307 (7.2)	< 0.0001
Valvular heart disease	151 (1)	87 (0.8)	64 (1.5)	< 0.0001
Other heart diseases	701 (4.6)	448 (4.1)	253 (5.9)	< 0.0001
Family history of stroke	2,476 (16.3)	1,474 (13.4)	1,002 (23.5)	< 0.0001
Current smoker	3,115 (20.5)	2,386 (21.7)	729 (17.1)	< 0.0001
Heavy alcohol consumption	2,526 (16.6)	2,010 (18.3)	516 (12.1)	< 0.0001
NIHSS on admission (mean ±SD)				
Ischemic stroke	9.6 ± 7.9	11.3 ± 8.0	6.2 ± 6.7	< 0.0001
Intracerebral hemorrhage	15.7 ± 9.5	16.2 ± 9.3	13.0 ± 10.1	< 0.0001
Subarachnoid hemorrhage	12.8 ± 13.0	13.6 ± 13.0	10.1 ± 12.7	0.0009
GCS on admission (mean ±SD)				
Ischemic stroke	13.5 ± 2.7	13.2 ± 2.8	14.0 ± 2.4	< 0.0001
Intracerebral hemorrhage	11.4 ± 3.9	11.4 ± 3.9	11.7 ± 3.9	0.0515
Subarachnoid hemorrhage	10.9 ± 4.5	10.8 ± 4.5	11.3 ± 4.6	0.1752
ICH score on admission of ICH cases (*n*, %)	(*n* = 4,715)	(*n* = 3,964)	(*n* = 751)	0.227
0	1,779 (37.7)	1,488 (37.5)	291 (38.7)	
1	1,119 (23.7)	937 (23.6)	182 (24.2)	
2	760 (16.1)	648 (16.3)	112 (14.9)	
3	584 (12.4)	493 (12.4)	91 (12.1)	
4	419 (8.9)	360 (9.1)	59 (7.9)	
5	46 (1)	35 (0.9)	11 (1.5)	
6	8 (0.2)	3 (0.1)	5 (0.7)	

GCS, Glasgow coma scale; ICH, intracerebral hemorrhage; NIHSS, National Institute of Health Stroke Scale; Php, Philippine Peso; SD, standard deviation.

Cases admitted in public hospitals were notably younger, more frequently male, unemployed or never worked, migrant from another region, and had lower annual income compared to those in private hospitals (all *p* < 0.0001). While known history or previous diagnosis of biological vascular risk factors, like hypertension, diabetes and dyslipidemia, was less frequent among cases in public hospitals, lifestyle risk factors, i.e., smoking and heavy alcohol consumption were more common. Stroke severity was different between public and private hospitals, with relatively more severe NIHSS in public hospitals.

### 3.3 Disparities in care between public and private hospitals

#### 3.3.1 Diagnostics

**Figure 2:**
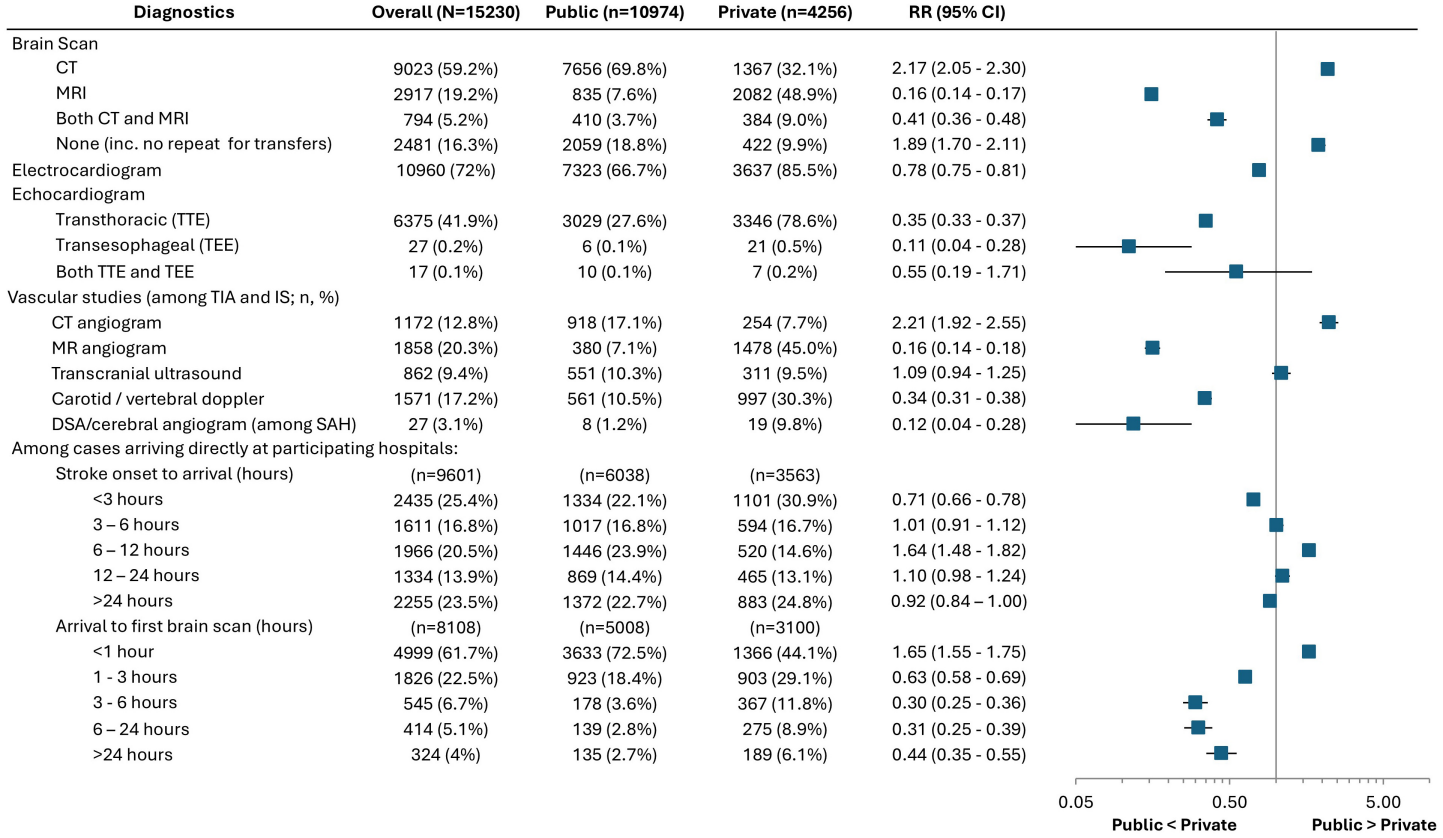
Diagnostic procedures performed on acute cerebrovascular cases in the PNA1DB-stroke overall and by type of hospital. CT, computed tomography; IS, ischemic stroke; MR, magnetic resonance; MRI, magnetic resonance imaging; SAH, subarachnoid hemorrhage; TEE, transesophageal echocardiogram; TIA, transient ischemic attack; TTE, transthoracic echocardiogram.

Computed tomography (CT) was the most common (59.2%) imaging technique used (Figure 2). Although electrocardiogram was performed in 10,960 (72%) of cases, only 6,419 (42%) had echocardiogram done. Likewise, the frequency of vascular studies was low. Among cases who presented directly at participating hospitals, median delay from stroke onset to hospital arrival time was 6.0 (IQR 2.5, 11.2) h with 2,435 (25.4%) cases arriving within 3 h and 2,255 (23.5%) arriving beyond 24 h. The median hospital arrival to brain imaging time was 0.8 (IQR 0.5, 2.0) h, with 4,999 (61.7%) cases being completed within 1 h.

The type of brain imaging performed was different between hospital types, with CT being more common in public hospitals and magnetic resonance imaging being more common in private hospitals. There were significantly less cardiac and vascular studies performed in public hospitals. While arrival in public hospitals within 3 h from stroke onset was less frequent compared to private hospitals (22.1% vs. 30.9%), brain imaging was performed within 1 h more often in public hospitals than in private hospitals (72.5% vs. 44.1%).

#### 3.3.2 Treatments

**Figure 3:**
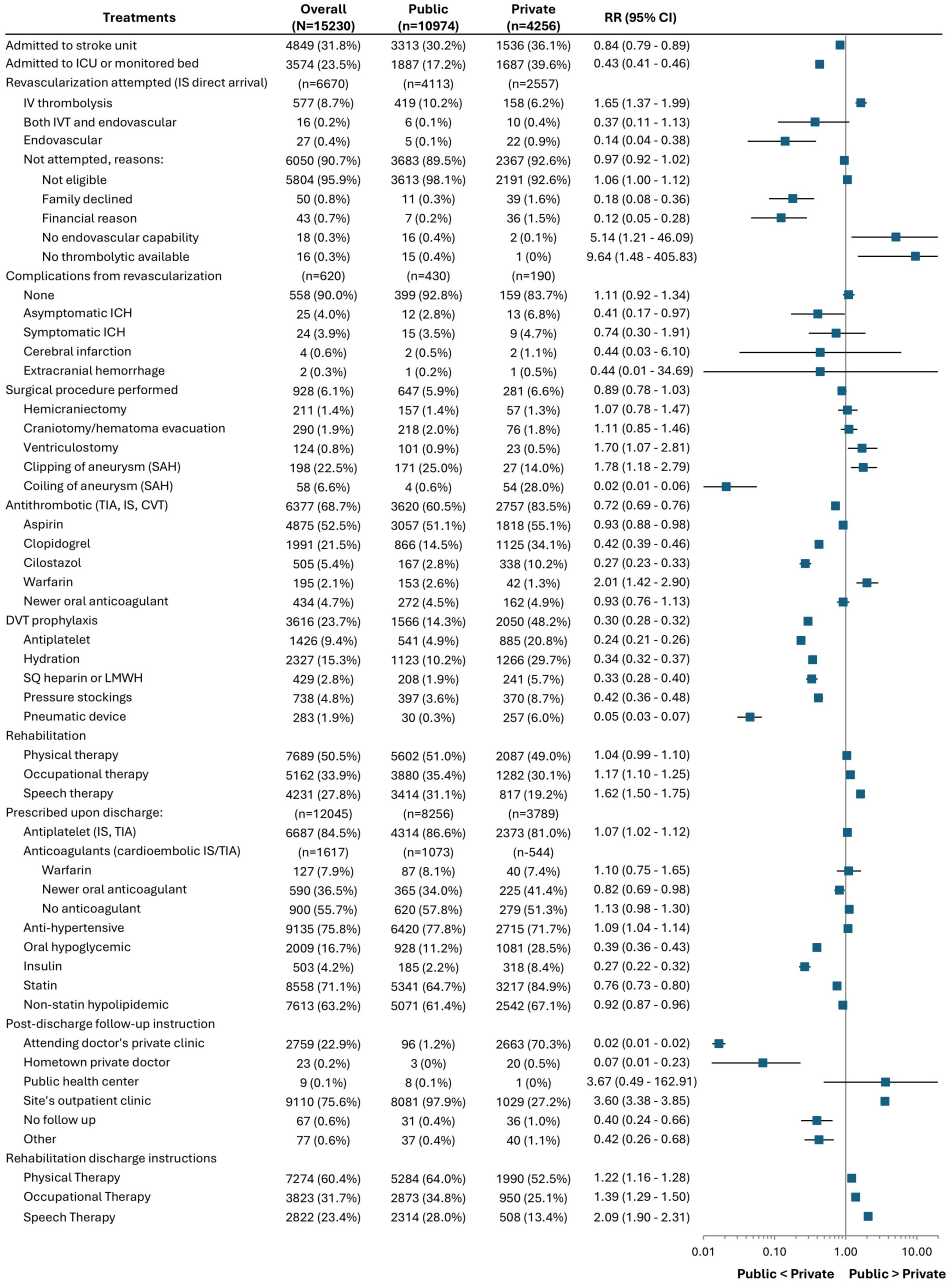
Treatments provided to acute cerebrovascular cases in the PNA1DB-stroke overall and by type of hospital. CVT, cerebral venous thrombosis; ICH, intracerebral hemorrhage; ICU, intensive care unit; IS, ischemic stroke; IV, intravenous; IVT, intravenous thrombolysis; LMWH, low molecular weight heparin; SAH, subarachnoid hemorrhage; SQ, subcutaneous; TIA, transient ischemic attack.

Only 4,849 (31.8%) were admitted to a stroke unit, while 3,574 (23.5%) were admitted to an intensive care unit or monitored bed (Figure 3). Of the 6,670 ischemic stroke cases who directly arrived at the participating hospital, only 620 (9.3%) had revascularization attempted by intravenous thrombolysis (IVT) and/or endovascular thrombectomy (EVT). The main reason for not attempting revascularization was non-eligibility (95.9%) that included arriving beyond the treatment window. While the overall rate of IVT among cases arriving within 4.5 h reached 27% (even up to 35.6% in public hospitals), the rates of EVT remained low among cases arriving within 24 h from stroke onset (Supplementary Table 2). Among those who underwent revascularization, 90% did not experience any complication, 4% had asymptomatic ICH, 3.9% had symptomatic ICH, 0.6% had recurrent cerebral infarction, and 0.3% had extracranial hemorrhage. Few cases underwent surgery, mainly for clipping (22.5%) and coiling (6.6%) of aneurysm among SAH cases.

During the patient's hospital stay, 68.7% were prescribed an anti-thrombotic medication, 23.7% given prophylaxis for deep venous thrombosis (DVT), and 50.5% received some form of rehabilitation service (Figure 3). Upon discharge, most (84.5%) of TIA and ischemic stroke cases were prescribed anti-platelets, while 55.7% of cardioembolic TIA or ischemic stroke cases did not receive any form of anticoagulation, although data were limited to clarify the reasons for non-anticoagulation. Most patients (75.6%) were instructed to follow up at the hospital's outpatient clinic and 60.4% were advised to undergo some form of rehabilitative therapy.

While cases were treated with intravenous thrombolysis more frequently in public hospitals, there were notably lower rates of admission to a stroke unit or monitored bed, attempts at endovascular thrombectomy, treatment with antithrombotic, and DVT prophylaxis (Figure 3 and Supplementary Table 3). Management of aneurysmal SAH was mainly surgical, i.e., clipping (25%), in public hospitals, while more often endovascular, i.e., coiling (28%) in private hospitals. Cases discharged from public hospitals were mostly (97.9%) instructed to follow up at the hospital's outpatient clinic, while those discharged from private hospitals were more often instructed to follow up in private clinics or in public health centers.

In multivariable models, observed disparities between public and private hospitals remained significant on many key diagnostic and main treatment procedures after adjustment for age, sex, (Table 2, model 1), and after further adjustment for stroke severity (model 2). In the fully adjusted model 3, age, sex, admission NIHSS, admission GCS, annual household income and delay in arrival time were not shown to be confounding drivers of acute management disparities. Many of these procedures involve the need for costly equipment and infrastructure.

**Table 2:** Multivariate models comparing public and private hospitals on rates of important diagnostic and treatment procedures.

Procedures	Adjusted *RR* (95% *CI*)		
	Model 1	Model 2	Model 3
Diagnostics			
Brain scan			
CT only	2.14 (2.04–2.24)	1,97 (1.88–2.06)	1.76 (1.66–1.86)
MRI only	0.16 (0.15–0.17)	0.22 (0.21–0.24)	0.28 (0.25–0.31)
Both CT and MRI	0.42 (0.36–0.48)	0.44 (0.38–0.51)	0.39 (0.33–0.47)
None (including no repeat upon transfer)	1.90 (1.72–2.11)	1.92 (1.72–2.12)	1.65 (1.42–1.90)
Electrocardiogram	1.00 (0.93–1.08)	1.00 (0.93–1.08)	1.00 (0.93–1.08)
Echocardiogram (TT or/and TE)	0.57 (0.56–0.59)	0.58 (0.56–0.59)	0.52 (0.50–0.53)
Vascular studies (among TIA and IS)	0.59 (0.57–0.61)	0.55 (0.53–0.58)	0.45 (0.43–0.48)
Among cases directly presenting to site			
Stroke onset to arrival < 3 h	0.46 (0.43–0.50)	0.43 (0.40–0.46)	0.95 (0.90–0.99)
Arrival to first brain scan < 1 h	0.72 (0.64–0.81)	0.65 (0.58–0.74)	0.85 (0.72–1.01)
In-hospital management			
Admitted to stroke unit or intensive care unit or monitored bed	0.69 (0.67–0.72)	0.70 (0.68–0.73)	1.02 (0.96–1.08)
Revascularization attempted (among direct arrivals, IS)			
IV thrombolysis	1.05 (0.88–1.25)	0.99 (0.81–1.20)	1.32 (1.02–1.72)
Endovascular	0.22 (0.13–0.37)	0.15 (0.08–0.26)	0.18 (0.10–0.35)
Antithrombotic (among TIA, IS, CVT)	0.72 (0.71–0.74)	0.79 (0.77–0.82)	0.86 (0.83–0.89)

Model 1: adjusted for sex and age; model 2: model 1 + admission NIHSS and admission GCS; model 3: model 2 + annual income, onset-to-arrival time/transfer status.

CT, computed tomography; CVT, cerebral venous thrombosis; IS, ischemic stroke; IV, intravenous; MRI, magnetic resonance imaging; NIHSS, National Institute of Health Stroke Scale; TE, transesophageal; TIA, transient ischemic attack; TT, transthoracic.

### 3.4 Outcomes

**Figure 4:**
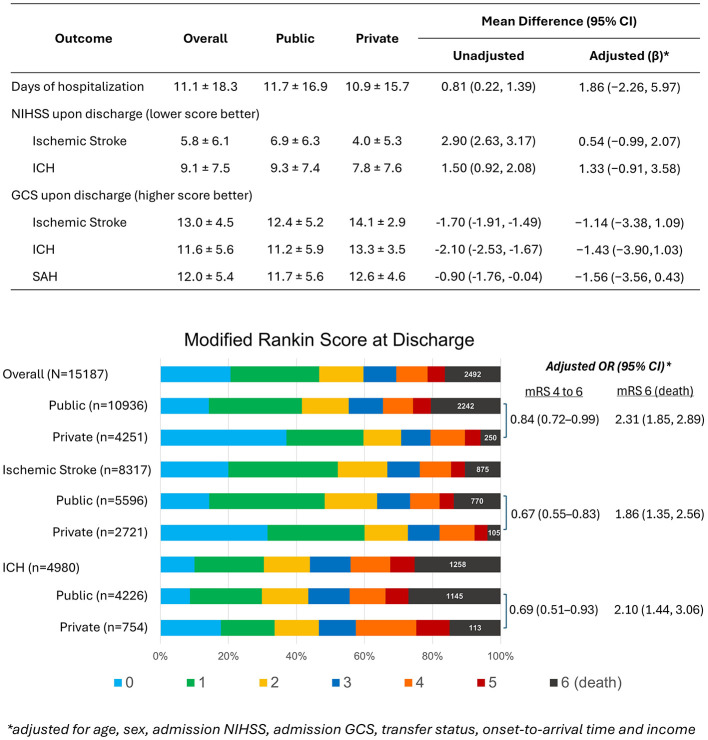
Length of hospital stay, National Institute of Health Stroke Scale (NIHSS), Glasgow Coma scale (GCS) and modified Rankin Scale at discharge of patients in PNA1DB-Stroke. ICH, intracerebral hemorrhage; SAH, subarachnoid hemorrhage; OR, odds ratio; Cl, confidence interval.

Median LOS of the study population was 5.0 (IQR 2.0, 10.5; mean 11.1 ± 18.3) days (Figure 4). Severity at time of discharge, as measured by NIHSS and GCS, was relatively worse for hemorrhagic strokes compared to ischemic stroke. Overall, in-hospital fatality rate was 16.4%. Compared to ischemic stroke, odds of death was worse for ICH (aOR 1.15, 95% CI 1.01–1.30).

While cases in public hospitals have longer hospital stay and worse NIHSS and GCS scores at discharge across stroke types compared to those admitted in private hospitals, the differences lost statistical significance after adjusting for age, sex, admission NIHSS and GCS, transfer status, onset-to-arrival time, and income, indicating important confounding by these factors. When severe functional dependency (mRS score 4–6) was analyzed, the adjusted odds ratio was in favor of public hospitals (*aOR* 0.84, 95% *CI* 0.72–0.99). In-hospital fatality (mRS score of 6), however, was higher in public hospitals overall and for either ischemic (*aOR* 1.86, 95% *CI* 1.35–2.56) or hemorrhagic stroke (*aOR* 2.10, 95% *CI* 1.44–3.06), suggesting that the cases with poorer prognosis in public hospitals tended to be fatal than surviving with severe functional dependency.

Among cases that receive revascularization therapies (*n* = 620), overall LOS was 8.6 ± 13.0 days, with no significant difference between those treated in public (9.0 ± 13.4 days) and private (7.7 ± 12.2 days) hospitals (*p* = 0.4457). Although improvement on NIHSS at discharge was better in private hospitals (2.1 ± 9.9 vs. 4.6 ± 8.9; *p* = 0.0016), achieving functional independence (mRS 0–1) was not significant different between public (*n* = 261, 60.7%) and private (*n* = 109, 57.4%) hospitals (*OR* 1.14 95% *CI* 0.81, 1.62).

## 4 Discussion

In this large multicenter cohort of 15,230 acute cerebrovascular cases across public and private hospitals in the PNA1DB-Stroke registry, we identified key patterns that underscore the complexity of stroke epidemiology, care, and outcomes in a lower-middle-income country (LMIC). Nearly 39% of all events were hemorrhagic, a proportion much higher than the 10%−15% reported in high-income countries but consistent with East Asian data, where intracerebral and subarachnoid hemorrhage account for approximately one-third of strokes (Yang et al., 2025; He et al., 2024; Satapathy et al., 2025). This regional similarity suggests shared epidemiologic and vascular risk profiles across Asian populations, particularly with respect to hypertension, underscoring the need for population-level blood pressure screening and treatment programs (Mills et al., 2020). Indeed, previous history of hypertension was the most common vascular risk factor reported in our study.

We observed an overall younger mean age of stroke onset compared with Japan, Singapore, and Western populations (Yang et al., 2025; Satapathy et al., 2025; The Lancet Regional Health—Southeast Asia, 2023; Matsuo, 2023). This shift toward working-age adults carries profound socioeconomic consequences, including reduced productivity and financial strain on households (Logan et al., 2024; Zhang et al., 2025). Global data demonstrate the incidence of young adult stroke to be rising faster in LMICs than in high-income countries (Collantes et al., 2022b; Satapathy et al., 2025; Béjot et al., 2016). While incidence and disability-adjusted life years have plateaued in high-income regions, they continue to rise in Southeast Asia (Feigin et al., 2024). This trend highlights the need for earlier identification and aggressive management of vascular and lifestyle risk factors in younger populations.

The disparities observed between public and private hospitals in the PNA1DB-Stroke were striking. As all participating hospitals were tertiary centers with accredited neurology training programs, hospital type and staff knowledge and skills were unlikely to independently account for the observed differences, but rather likely due to differences in case mix, referral pattern, resources, and patient financial capacity.

Public hospitals admitted younger, socioeconomically disadvantaged patients who were more often transferred from another hospital and relatively more severe. They were more likely to smoke and consume alcohol, but less likely to have known histories of hypertension, diabetes, or dyslipidemia. These differences suggest underdiagnosis and late healthcare engagement rather than true differences in prevalence, indicating gaps in primary care and preventive screening (Collantes et al., 2021; Logan et al., 2024). In contrast, private hospitals admitted older, wealthier patients who may have better access to regular medical care. Similar inequities have been described in other LMICs, where delayed diagnosis of vascular risk factors was associated with higher first-stroke severity and worse outcomes (Owolabi et al., 2022). Importantly, these findings suggest that social determinants of health may substantially influence stroke recovery independent of acute treatment delivery.

One-quarter of cases in our cohort were transferred from another hospital, most often from smaller private or less-equipped public facilities to participating government hospitals. Several factors may explain this high transfer rate. First, financial constraints likely drove patients initially seen in private hospitals to seek definitive care in public institutions where costs are subsidized, a pattern described in other LMICs as well (Collantes et al., 2022b; Webster et al., 2016). Second, the need for higher-level interventions, particularly neurosurgical procedures for hemorrhagic strokes, may have necessitated transfer to government tertiary centers with surgical expertise and critical care capacity (Adeoye et al., 2019). Third, the uneven distribution of stroke-ready hospitals and the scarcity of neurointerventional facilities in the Philippines likely contribute to dependence on referral pathways. Such transfers introduce treatment delays, particularly for time-sensitive therapies such as thrombolysis and thrombectomy, and may account for the huge proportion of our cases being ineligible for such acute therapies (Hassan et al., 2022; Chung et al., 2023).

Resource disparities further shaped patient pathways, although differences in patient profiles between public and private hospitals may influence resource utilization. While head CT scan was widely available, advanced imaging, vascular studies, and cardiac evaluations were less frequently utilized in public hospitals. These limitations may restrict identification of underlying etiologies necessary for optimizing secondary prevention strategies (Collantes et al., 2022b; The Lancet Regional Health—Southeast Asia, 2023). Only 9% of ischemic stroke patients underwent revascularization mainly due to eligibility issues and very low rate of neurointervention. Endovascular thrombectomy (EVT) remain concentrated in few tertiary centers. Among the 11 participating hospitals, only one of the five public hospitals and three of the six private hospitals were EVT-capable. Admission to stroke units and or monitored bed was also generally lower in public hospitals despite having more severe cases. These findings echo prior reports across Southeast Asia that described insufficient stroke unit coverage and inequitable access to evidence-based interventions (Collantes et al., 2022b).

Another important consideration is the role of financial cost in influencing treatment, particularly as regards reperfusion therapies. Recombinant tissue plasminogen activator (r-tPA) was recently provided free of charge in public hospitals under government subsidy programs, whereas patients admitted to private hospitals typically incur the substantial out-of-pocket expense particularly among those without comprehensive health insurance. This financial barrier may partly explain why r-tPA use was paradoxically more frequent in public hospitals despite fewer patients arriving within 3 h of stroke onset. Financial reason for declining treatment was less commonly reported compared with private hospitals. Prior reports from LMICs indicate that direct treatment costs may be a major obstacle to timely reperfusion, even when patients present within the therapeutic window (Gajurel et al., 2023; Opare-Addo et al., 2023). Hence, expanding national health insurance coverage to include reperfusion therapies across both public and private settings may help ensure equitable access (Cordero, 2022).

Apparent disparities in clinical outcomes upon discharge between public and private hospitals were observed. Although cases treated in public hospitals experienced longer hospital stays, worse neurological status at discharge, and higher in-hospital fatality, attenuation of the differences after adjustment for confounders indicates that outcomes were likely affected by dissimilarities in case profile, clinical severity, and the large proportion of cases transferred to public tertiary centers. International data demonstrated interhospital transfer to be often associated with treatment delays and reduced effectiveness of reperfusion therapies (Hassan et al., 2022; Katsanos et al., 2024). These findings highlight the urgent need for capacity-building in more strategic hospitals and development of coordinated stroke networks. Streamlined inter-facility transfer protocols and the integration of telemedicine, as seen in mature stroke systems of care, may help further reduce preventable delays and potentially improve outcomes (Hassan et al., 2022; de Andrade et al., 2025).

Taken altogether, our study using markers of quality of care in selected public and private tertiary hospitals as proxies reveals how socioeconomic factors, clinical profile and health system fragmentation may shape stroke care and outcomes, reflecting the broader global divide between high-income and LMICs (Satapathy et al., 2025; Logan et al., 2024; Feigin et al., 2024). The 2025 American Heart Association Heart Disease and Stroke Statistics report similarly emphasized persistent inequities worldwide and called for equity-focused solutions (Martin et al., 2025).

This study has several important limitations that should be considered when interpreting the findings. As a hospital-based registry, it may not fully capture community stroke events, particularly patients who never reached medical care, potentially biasing estimates (Strong et al., 2007). While the participating centers were major accredited neurology training hospitals, the registry may have over-represented patients from urban areas and those with more severe strokes who were transferred and managed under specialist neurological services. Although most cases had imaging confirmation, a small subset lacked brain imaging due to early death, discharge, or financial constraints. Differences in diagnostic availability across hospitals may have led to underestimation of certain stroke subtypes and mechanisms. Post-discharge outcomes were not captured, limiting assessment of long-term disability and recovery (Diestro et al., 2021). Socioeconomic and comorbidity data relied on patient or caregiver reports and may have introduced recall bias. Finally, as an observational registry study, residual confounding especially from referral patterns and unmeasured social determinants of health, cannot be excluded (von Elm et al., 2008). Causal links and effectiveness of interventions are difficult to establish. Nevertheless, the extensive data from consecutive cases and multicenter design encompassing both public and private hospitals provide a comprehensive overview of the real-world condition on stroke care and highlight key potential priority areas for reforms.

Thus far, our findings point to critical opportunities to strengthen and restructure stroke care by directing support and resources where they are needed. The high burden of hemorrhagic stroke and younger age of onset suggest a need to emphasize prevention strategies by early detection and control of risk factors, particularly for middle-aged adults. Underdiagnosis of comorbidities in public hospitals reflects gaps in primary care, and equitable access to diagnostics, therapies, and rehabilitation may improve with expansion of services through subsidies and improved financing. At the national level, substantial work remains in improving the quality of stroke care, especially in rural and underserved areas, by identifying where the gaps are and addressing them. The PNA and Stroke Society of the Philippines have been central in developing locally relevant guidelines (Stroke Society of the Philippines, 2025). Yet, their implementation is constrained by economic, infrastructural, workforce, and socio-cultural barriers. The PNA1DB-Stroke registry offers a foundation for identifying needs and directing resource allocation, and could serve as a model for stroke-ready networks nationwide. Closing the gap between evidence and practice will require data-driven and context-specific strategies to address the barriers and monitor successful implementation. Without reforms, equitable and timely stroke care will remain out of reach; with them, it can become the national standard (Feigin and Owolabi, 2023).

## Data Availability

The original contributions presented in the study are included in the article and supplementary materials. Reasonable requests for specific individual data for legitimate purposes may be sent to the corresponding author, robert_gan2@hotmail.com.

## Group members of the Philippine Neurological Association One Database for Stroke

Study Organization

PNA1DB Project Steering Committee—Rosalina E. Picar (Chair); Data Manager—Jose Leonard R. Pascual V; Project Manager—Oliber Biaro; Data Completion Monitor—Neil Manro Planas.

PNA1DB-Stroke Disease Study Management Group: Robert N. Gan (Chair), Raquel M. Alvarez, Maria Teresa A. Cañete, Christian Oliver C. Co, Maria Epifania V. Collantes, Cyrus G. Escabillas, John Harold B. Hiyadan, Dan Neftalie A. Juangco, Johnny K. Lokin, Ma. Cristina Z. Macrohon-Valdez, Belinda L. Mesina-Nepomuceno, Monique Therese S. Punsalan (former), Gemmalynn B. Sarapuddin.

PNA1DB-Stroke hospitals and contributors: Baguio General Hospital (2,423)—J. H. B. Hiyadan, G. T. Gayo, M. D. Liban, R. Ulpindo, R. San Juan, T. Banisa, Y. Fontanilla; Chong Hua Hospital (679)—M. T. A. Cañete, M. Eribal, J. F. Rabor, S. Im, A. Osayan, K. Solijon, G. Cabatana, K. Panares; East Avenue Medical Center (2,007)—D. N. A. Juangco, V. Sambale II, R. A. M. Carandang, J. A. Montoya, E. Montemayor, M. F. Isidoro, K. R. Ang, J. A. Tee, P. Demaisip, C. Rabadon, M. M. Cambalon, F. Almelor, J. A. Chua, J. C. Ruiz, L. A. Danganan, J. T. Sydiongco, M. K. Hernandez, J. A. Surtida, G. V. Aguas, R. L. Bautista, C. N. Fernan, R. J. Jecino II, F. G. Pua, D. Talavera, J. M. Pleños, J. M. Nombre, T. Dumo-Buenaventura, A. Graza, M. L. Infante, A. Cepillo, R. Ulep, A. Echon, A. Caballero, M. J. Incognito; Jose R. Reyes Memorial Medical Center (1,184)—C. G. Escabillas, J. C. Navarro, R. U. Esagunde, M. J. Torrefranca, M. P. T. Ogena, J. C. V. Roque, A. De Leon, M. T. T. Cinco, L. K. J. Batino, A. P. R. De Leon, P. N. Gonzales, M. E. Ramos, Z. F. A. Sabellano, A. D. Navarro, L. M. L. Llapitan, M. F. K. Reyes; Makati Medical Center (677)—R. M. Alvarez, P. K. Jaca, J. P. Delfino, J. A. Gamboa, M. A. Sarmiento, J. M. Geotina, L. J. E. Arcenas, K. Bellosillo, C. C. Matute, F. P. Padilla, K. P. Amamanglon, N. K. Alambra, J. L. Tuason, A. Escultero, L. S. Balanquit, M. F. Cabalu, G. Olayan, K. N. Batalla, K. Puzon, M. S. Dating, J. Jumalon, C. J. Osia, C. Y. Macias, J. Maranan; Philippine General Hospital (4,182)—M. E. V. Collantes, J. L. Pascual, K. Separa, M. C. S. Lukban, M. E. Gregorio, L. P. Dichoso, S. Guinal, M. J. Arada, P. A. Armamento; Quirino Memorial Medical Center (1,178)—CO Co, M. A. Pura, A. I. J. Pajantoy, K. K. Ompoc, J. P. Cruz, A. Pasiwat, R. Jr. Galvan, J. M. Calubayan, M. R. Rebanal-Ricafrente, J. C. Saballegue, M. F. Pinto, R. M. O. Tan, C. J. S Quanico, A. Y. M. Nietes, E. Dela Torre; St. Luke's Medical Center (1,396)—M. C. Z. Macrohon-Valdez, J. L. R. Pascual, M. I. Escasura, D.-L. Que, P. Yalung, M. D. Mascareñas, A. J. A. Bacarra, E. Honrado, M. J. D. Francisco, J. L. H. Soller, J. F. Beloy, J. B. Canas, J. Tapia, M. J. Bitang, C. A. Caccam, D. G. Feria, J. Sta. Cruz, E. K. Delos Santos, K. Z. Alviar, R. Sadie, Z. Francisco, M. Ogalesco, E. F. Caro, J. Templonuevo; The Medical City (886)—G. B. Sarapuddin, M. J. S. Amoranto, J. M. Piawan, K. Geronimo, P. K. P. V. Quintay, A. R. Olano, E. T. D. Villoria, M. Fuentes, G. A. Constantino, G. G. Garrido-Bagayo, K. A. E. Inting, A. J. A. Yumang, J. E. A. Corpuz, C. M. P. Guerrero, S. Guerra, R. O. Alinab, K. G. Lipardo, L. M. Alcantara, R. U. Tan; Universtiy of the East-Ramon Magsaysay Memorial Medical Center (347)—B. L. Mesina-Nepomuceno, M. T. S. Punsalan, D. Q. Perez, T. F. B. Reyes, L. M. J. Bagares, R. A. Sususco, M. C. B. Nunez, P. R. P. Mejia, G. N. P. Navata, K. C. Dayrit, A. A. R. Sanchez, A. M. U. Chua, K. Po, A. J. Sy, Y. J. Rabe, C. J. Navarra, L. C. Alba; University of Santo Tomas Hospital (271)—J. K. Lokin, J.-A. Catindig, A. Baroque, J. Evangelista, G. A. Baroque, N. Endaya, M. A. De Leon, S. K. Inoncillo, M. J. Trivilegio.
