# Supplementary material for: Acute cerebrovascular disease in the Philippine Neurological Association One Database (PNA1DB)—patient profiles and disparities between public and private hospitals
Source: Front Stroke. 2026 Jun 29;5:1835562. doi: 10.3389/fstro.2026.1835562 (PMC13356936; doi:10.3389/fstro.2026.1835562)
Supplement: Supplementary file 1 [file Table_1.docx]

Supplement Table 1. Missing data of key variables, n (%).

| Variables | Overall  (N=15230) | Public  (n=10974) | Private  (n=4256) |
| --- | --- | --- | --- |
| Annual household income (incl. declined to answer) | 653 (4.3%) | 11 (0.1%) | 642 (15.1%) |
| Occupation (incl. declined to answer) | 2552 (16.7%) | 2123 (19.3%) | 429 (10.1%) |
| No brain imaging data | 76 (0.5%) | 69 (0.6%) | 7 (0.2%) |
| Ischemic stroke arriving directly at hospitals | (n=6670) | (n=4113) | (n=2557) |
| Stroke onset to arrival time (unable to compute)* | 194 (2.9%) | 71 (1.7%) | 123 (4.8%) |
| Arrival to first imaging (unable to compute)** | 370 (5.5%) | 238 (5.8%) | 132 (5.2%) |
| No NIHSS on admission | 9 (0.1%) | 1 (0.0%) | 8 (0.3%) |
| No GCS on admission | 29 (0.4%) | 3 (0.1%) | 26 (1.0%) |
| No revascularization data | 0 (0.0%) | 0 (0.0%) | 0 (0.0%) |
| No discharge NIHSS (including dead) | 65 (1.0%) | 54 (1.3%) | 11 (0.4%) |
| No discharge mRS | 4 (0.1%) | 1 (0.0%) | 3 (0.1%) |
| Cardioembolic ischemic stroke or TIA | (n=1292) | (n=756) | (n= 536) |
| No data on discharge anticoagulation | 114 (8.8%) | 43 (5.7%) | 71 (13.2%) |

*Missing stroke onset time or arrival time; **missing arrival time or brain imaging time

GCS= Glasgow Coma Scale, NIHSS= National Institute of Health Stroke Scale, mRS= modified Rankin Scale, TIA= transient ischemic attack

Supplement Table 2. Rates of revascularization among ischemic stroke cases arriving directly at participating hospitals by onset to arrival time, n (%).*

| Stroke onset to arrival time | Overall | Public | Private |
| --- | --- | --- | --- |
| Within 4.5 hours |  |  |  |
| IVT | 454 (27.5%) | 317 (35.6%) | 137 (18.0%) |
| EVT (± IVT) | 24 (1.4%) | 5 (0.5%) | 19 (2.5%) |
| Between 4.5 to 6 hours |  |  |  |
| EVT (± IVT) | 2 (0.4%) | 2 (0.6%) | 0 (0.0%) |
| Between 6 to 24 hours |  |  |  |
| EVT (± IVT) | 10 (0.4%) | 2 (0.2%) | 8 (1.1%) |

*Cases included if both stroke onset and arrival times were reported

IVT= intravenous thrombolysis, EVT= endovascular thrombectomy

Supplement Table 3. Frequency and methods of DVT prophylaxis according to event type, n (%). Note: more than one method may be used in a case.

| Event type and Method | Overall | Public | Private |
| --- | --- | --- | --- |
| Ischemic Stroke (any method) | 2338 (28.0%) | 984 (17.5%) | 1354 (49.7%) |
| Antiplatelet | 1214 (14.6%) | 503 (9.0%) | 711 (26.1%) |
| Hydration | 1458 (17.5%) | 647 (11.5%) | 811 (29.8%) |
| SQ heparin or LMWH | 360 (4.3%) | 159 (2.8%) | 201 (7.4%) |
| Pressure stockings | 417 (5.0%) | 199 (3.5%) | 218 (8.0%) |
| Pneumatic device | 140 (1.7%) | 9 (0.2%) | 131 (4.8%) |
| Intracerebral hemorrhage (any method) | 860 (17.2%) | 485 (11.4%) | 375 (49.7%) |
| Antiplatelet | 21 (0.4%) | 16 (0.4%) | 5 (0.7%) |
| Hydration | 649 (13.0%) | 397 (9.4%) | 252 (33.4%) |
| SQ heparin or LMWH | 51 (1.0%) | 35 (0.8%) | 16 (2.1%) |
| Pressure stockings | 292 (5.8%) | 168 (4.0%) | 124 (16.4%) |
| Pneumatic device | 111 (2.2%) | 19 (0.4%) | 92 (12.2%) |
| Subarachnoid hemorrhage (any method) | 149 (17.0%) | 67 (9.8%) | 82 (42.5%) |
| Antiplatelet | 9 (1.0%) | 6 (0.9%) | 3 (1.6%) |
| Hydration | 107 (12.2%) | 61 (8.9%) | 46 (23.8%) |
| SQ heparin or LMWH | 19 (2.2%) | 11 (1.6%) | 8 (4.1%) |
| Pressure stockings | 44 (5.0%) | 27 (3.9%) | 17 (8.8%) |
| Pneumatic device | 28 (3.2%) | 2 (0.3%) | 26 (13.5%) |

DVT= deep venous thrombosis, SQ= subcutaneous, LMWH= low molecular weight heparin
